# 

**Published:** 1887-10-29

**Authors:** 


CONTENTS.

**Women Doctors for Indian Women** - - - 71

**Words of Consolation.**—Chapter LVI. The Sufferings of Christ - - - - - - 72

**The Hospitals and the Public.** By BARON FERDINAND DE ROTHSCHILD, M.P. - - - - - - 73

**The Registration of Nurses** - - - - - 74

**The Doctor's Pulpit.**—Scarlet Fever—The Dangers of Convalescence - - - - - - 75

**The Common Accidents of Every-day Life.**—By R. A. P. FORRESTER, B.A. - - - - - 76

**Workpeople's Subscriptions** - - - - - 76

**Professional Notes.** By a PHYSICIAN - - - - 77

**Notes and News.**—Miscellaneous Items.—The Whale at Tilbury Fort.—The Hospitals Association Meetings.—Vacancies.—Condensed Milk.—Dearth of Doctors in Russia.—The Scarlet Fever Epidemic.—The Venus of Milo, etc., etc. - 78

**Annotations.**—Lady Doctors in France.—The Dangers of the Dentist's Chair.—Truth and Justice—and Houses to Let - 81

**Medicine Men of the World.** By the MAN IN THE CROWD.—II. Medicine Men in Savage Lands - - - 82

**Sick Nursing in the Sixteenth Century.** By a STUDENT OF MEDIÆVAL INSTITUTIONS - - - - 83

**Liquid Foods** - - - - - - - - 84

**East and West.** By LESLIE KEITH.—Chapter V. A Poet in London - - - - - - - 85

**The Matrons' Corner.**—A Lady Cook.—The Health of Probationers - - - - - - - 87

**Small-pox at the Perth Infirmary** - - - - 87

**Hospitals, Doctors, Treatment, and Attendance.** The In- and Out-Patients' Hospital Diary - - - 88

**Amusements with Prizes, for all Ages.**—For Men and Women.—The Children's Corner.—Theatres and Concerts iv

